# Comparing trends in the use and outcomes of non invasive ventilation (NIV) in a general intensive care unit

**DOI:** 10.1186/2197-425X-3-S1-A675

**Published:** 2015-10-01

**Authors:** L Wilson, J Gross, G Gallagher, A Wolff

**Affiliations:** Royal Free London NHS Foundation Trust, Intensive Care Unit, Barnet Hospital, London, United Kingdom

## Introduction

There has been an increase in the use of non-invasive ventilation (NIV) in the intensive care setting. Guidelines suggest more favourable outcomes when used in patients with chronic obstructive pulmonary disease (COPD) and cardiogenic pulmonary oedema (CPO)[1], yet its use often extends beyond these indications despite no evidence of clear benefit.

## Objectives

To review the practice and outcomes of NIV in our institution over a 1 year period.

## Methods

We conducted a retrospective analysis of all patients receiving NIV for the first time at any stage of their ICU admission between 1^st^ January 2014 and 31^st^ December 2014. Patients were divided into 3 groups: decompensated respiratory failure (DRF) (pH< 7.35 + pCO2>6KPa pre-NIV), non-decompensated respiratory failure (NDRF) (pH>7.35 or PCO2< 6Kpa pre-NIV) or post extubation. For each patient, the indication and duration of NIV was recorded along with important outcomes that included requirement and duration of mechanical ventilation (MV), length of ICU stay and survival to ICU discharge.

## Results

**Figure 1:**
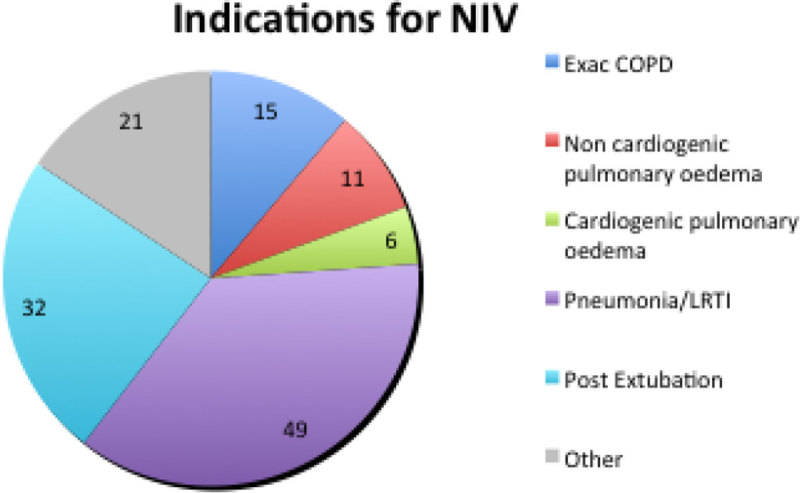
**Indications for NIV**

103 patients received NIV as a first line respiratory supportive therapy for a number of indications of which only 20% included COPD or CPO (figure 1).

38 had DRF and 63 NDRF prior to initiation of NIV. Despite those with DRF having significantly higher predicted acute hospital mortality than NDRF according to admission ICNARC scoring (49% [IQR 28-62] vs. 36% [IQR 10-54] p = 0.017), median duration of NIV received was similar (23 [IQR 11-105] and 17 [IQR 8-73.5] hours for DRF and NDRF respectively (p = 0.13)). There were no significant differences between DRF and NDRF with respect to % of patients requiring subsequent mechanical ventilation (29% v 43%; p = 0.16), median duration of mechanical ventilation for those subsequently intubated (8 [IQR 4-11] vs. 9 [IQR 6-18] days; p = 0.76) or % surviving to ICU discharge (71% v 71%; p = 0.97). There was a non-significant trend towards reduction in ICU length of stay (LOS) for those with DRF compared with NDRF (median ICU LOS 5.2 [IQR 3.1-8.9] v 9.8 [IQR 4.7-12.8] days respectively; p = 0.052).

NIV following a period of mechanical ventilation was given for 31 patients. In this cohort, NIV was administered for a median duration of 21 hours (range 1-617 hours). 13/31 patients (42%) required re-intubation and further periods of mechanical ventilation.

## Conclusion

In our institution, the majority of patients received NIV for indications outside those recommended by guidelines. Those with DRF receiving NIV fared no worse compared with NDRF in this cohort. This along with those receiving NIV following extubation may provide a cohort of patients who warrant further investigation.
